# Dietary Management After Ulcerative Colitis Surgery: A Thematic Analysis of TikTok Content

**DOI:** 10.3390/nu18071110

**Published:** 2026-03-30

**Authors:** Oliver R. Kaye, Dakota R. Rhys-Jones, Orestis Argyriou, Sue Blackwell, Emma P. Halmos, Zaid Ardalan, Janindra Warusavitarne, Kapil Sahnan, Jonathan P. Segal, Ailsa L. Hart, Chu K. Yao, Itai Ghersin

**Affiliations:** 1 Faculty of Medicine and Health Sciences, University of East Anglia, Norwich NR4 7TJ, UK; oliverrkaye@hotmail.co.uk; 2 Department of Gastroenterology, School of Translational Medicine, Monash University, Melbourne 3004, Australia; dakota.rhys-jones@monash.edu (D.R.R.-J.); emma.halmos@monash.edu (E.P.H.); zaid.ardalan@gmail.com (Z.A.); chu.yao@monash.edu (C.K.Y.); 3 Department of Colorectal Surgery, St Mark’s National Bowel Hospital & Academic Institute, London NW10 7NS, UK; orestis.argyriou@nhs.net (O.A.); j.warusavitarne@nhs.net (J.W.); kapil.sahnan@nhs.net (K.S.); 4 Department of Surgery and Cancer, Imperial College London, London W12 0NN, UK; 5 Institute of Applied Health Research, University of Birmingham, Birmingham B15 2TT, UK; sueblackwell5@gmail.com; 6 Department of Gastroenterology, Royal Melbourne Hospital, Melbourne 3052, Australia; segaljonathan0@gmail.com; 7 Department of Medicine, University of Melbourne, Parkville 3050, Australia; 8 Department of Gastroenterology, St Mark’s National Bowel Hospital & Academic Institute, London NW10 7NS, UK; ailsa.hart@nhs.net; 9 Department of Metabolism, Digestion and Reproduction, Imperial College, London W12 0NN, UK; 10 Department of Gastroenterology, Eastern Health Clinical School, Eastern Health & Monash University, Melbourne 3128, Australia

**Keywords:** colectomy, diet, ileo-anal pouch, nutrition, social media, ulcerative colitis

## Abstract

**Background/Objectives**: For patients with Ulcerative Colitis (UC) requiring surgical treatment, post-operative dietary management can pose significant challenges. TikTok is emerging as a popular social media platform for dissemination of health and nutrition information. The aim of this study is to analyse patient-generated content on TikTok regarding dietary management post-UC surgery, in order to identify recurring themes and highlight patient priorities. **Methods:** Relevant TikTok videos were identified through a systematic search. Search terms were developed by combining ‘diet UC’ or ‘nutrition UC’ with common UC surgical procedures. From each search term, the first 10 videos were screened. If a search produced fewer than 10 results, all identified videos were retrieved. Inclusion criteria were videos in English, and a strong indication that the content creator was diagnosed with UC and had undergone relevant surgery, and was providing nutrition recommendations. Thematic analysis of video transcripts was conducted using Braun and Clarke’s framework to identify common themes. **Results:** A total of 89 videos, created between 2021 and 2024, were found on the initial search, of which 12 duplicates were removed, and 77 videos were screened. Sixteen English language videos met the inclusion criteria and were analysed. Thematic analysis identified three overarching themes: (1) *adaptive dietary progression in the post-surgical period*, where patients described a phased approach to reintroducing foods post-surgery; (2) *personalisation of diet*, highlighting individualised strategies for symptom and hydration management; and (3) *Emotional and social impact of dietary restrictions and modifications*, including fear of food and social isolation. **Conclusions:** This thematic analysis offers an insight into how patients navigate the complex management of diet following UC surgery. It is important for clinicians to discuss the dietary information and online content patients are exposed to in relation to their condition. Additionally, clinical practice should evolve to embrace patient-centred, multidisciplinary approaches that validate lived experience, ensure consistent dietary guidance, and address the psychological burden of dietary restriction.

## 1. Introduction

Ulcerative Colitis (UC) is a chronic, immune-mediated disease characterised predominantly by mucosal inflammation and ulceration of the colon [1]. Although UC can present at any age, most patients are diagnosed in the second or third decade of life [1]. Despite advancements in medical therapies for UC, about 15% of patients will require a colectomy 10 years after their diagnosis [2]. Post-colectomy surgical options include restorative options such as an ileo-anal pouch anastomosis (IPAA) or an ileorectal anastomosis (IRA) or an ileostomy [3].

Managing diet post-surgery can be challenging for patients. The absence of the colon results in increased stool frequency, reduced fluid absorption, and a change in stool consistency [4]. Mitigating against these requires tailored dietary strategies to manage symptoms, maintain hydration, and optimise nutritional status [4]. Although assessment from specialised dietitians is recommended by international guidelines [5], access to these services is not always available. Patients are often left to self-experiment, which can lead to unnecessarily over-restricting their diet, and in some cases can even lead to negative coping strategies [6,7]. Concerningly, even when given dietary advice from health professionals, patients have reported inconsistent dietary advice and a lack of personalised guidance on what and how to eat [7].

Due to the apparent lack of dietary guidance from healthcare professionals, an increasing number of UC patients are seeking dietary advice online. Unfortunately, a recent quality assessment of credible online dietary resources conducted by our group for patients with an ileo-anal pouch found these online resources were of poor quality and difficult to understand [8]. Social media platforms, such as TikTok, are increasingly popular for patients seeking health information [9], particularly for patients managing chronic illnesses, such as UC. TikTok’s algorithm-driven engagement creates a unique environment where shared patient experiences can be widely accessed and amplified by medical professionals beyond their traditional follower networks. Unlike other platforms such as Instagram or Twitter/X, an established following is not needed for content to have wide reach, a feature which is particularly popular with younger users [10].

However, TikTok is largely unregulated and driven by algorithm-based engagement, which may expose patients to popular yet inaccurate or misleading health and nutrition content [11,12]. Moreover, evidence suggests that TikTok content can shape young people’s eating attitudes and body image, frequently promoting a negative diet culture, body dissatisfaction, and eating disorder-related messages, highlighting the platform’s potential to contribute to unhealthy eating behaviours in this population [13,14,15]. Understanding the types of content patients commonly encounter may help clinicians better identify patient priorities and address dietary information needs following UC surgery.

Therefore, the aim of this study is to analyse patient-generated content on TikTok regarding dietary management post-UC surgery. We chose to specifically focus on TikTok due to its particular popularity among younger patients.

## 2. Materials and Methods

In order to minimise algorithmic bias, and prevent prior search history from influencing video suggestions, a new TikTok profile was created exclusively for this study.

A preliminary search using the discovery function was undertaken using various combinations of key words and hashtags related to UC and nutrition/diet that would generate relevant and popular videos. This process generated a large list of videos associated with multiple search terms. Based on preliminary exploration and iterative discussion, the list of hashtags and keywords was refined to develop a more systematic and targeted search strategy. Search terms were developed by combining ”diet UC” or ”nutrition UC” with specific surgical procedures, resulting in the following ten search combinations:

(1) ileostomy diet UC.
(2) ileostomy nutrition UC.
(3) stoma diet UC.
(4) stoma nutrition UC.
(5) J pouch nutrition.
(6) J pouch diet.
(7) ileorectal UC nutrition.
(8) ileorectal UC diet.
(9) continent ileostomy UC nutrition.
(10) continent ileostomy diet UC.

Inclusion criteria were as follows:

(1) Videos in the English language.
(2) Strong indication that the content creator has UC and underwent surgical treatment for it.
(3) Discussion of nutrition/diet.

Videos were excluded if they were:

(1) In a language other than English.
(2) Referencing UC without any mention of surgery.
(3) Discussing Crohn’s disease, bowel cancer, or any other non-UC gastrointestinal condition.
(4) Not discussing nutrition/diet.

Data extraction followed a standardised protocol: The top 10 videos generated by each search query were captured in an Excel spreadsheet. If a query produced a total volume of fewer than 10 results, all identified videos were retrieved to ensure maximum coverage of that specific search term.

The search was conducted on a single date (13 March 2025) and video links were saved.

All videos were transcribed using an online tool (https://www.script.tokaudit.io/). We used V2 of this tool. It was last accessed on 10 April 2025. This was followed by a thematic analysis of the video content to get a deeper understanding of patient experiences across the dataset. This flexible type of analysis was informed by Braun and Clarke’s principles of thematic analysis [16], which involves familiarisation with data and identifying patterns in qualitative data. Briefly, videos were first watched individually by members of the research team without taking notes. In the second round, researchers then watched the videos together, discussed initial ideas of emerging themes, and documented notes. Transcripts were then analysed and quotes were grouped according to each theme.

Descriptive results of video engagement were also recorded and reported.

## 3. Results

**Figure 1:**
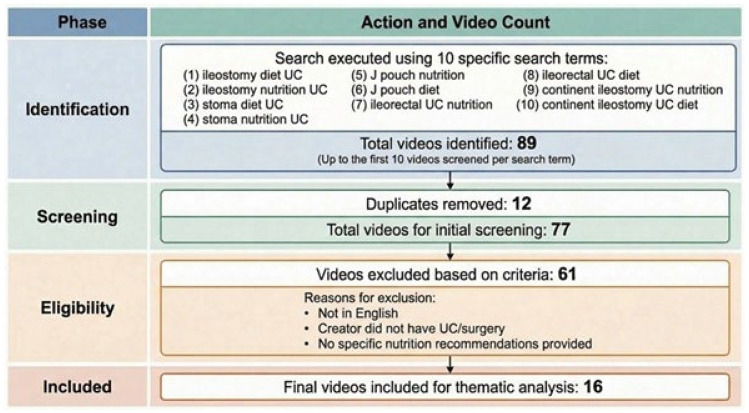
Flow diagram of TikTok video selection for thematic analysis.

Our initial search yielded 89 videos, of which 12 duplicates were removed, and 77 videos were screened. Of the 77 videos which were screened, 16 videos met the inclusion criteria, as outlined in Figure 1. These videos were created between 2021–2024, and countries of origin varied from the UK, USA, Canada, and Morocco, as outlined in Table 1. Of the 16 content creators, 12 were patients with lived experience, 3 were health professionals (1 colorectal surgeon, 1 dietitian, and 1 nurse), and 1 was a stoma bag company.

Engagement metrics varied widely, with the range of likes for each video being between 16–1175 (mean = 203) at time of access, and total account followers ranging from 110–1.9M (mean = 19,960).

Table 1 describes the characteristics of each account. Thematic analysis: Our analysis identified three overarching, interconnected themes:

(1) Adaptive dietary progression in the post-surgical period.
(2) Personalisation of diet for symptom and hydration management.
(3) Emotional and social dimensions of dietary restrictions and modifications.

### 3.1. Dietary Adaptations in the Post-Surgical Period

A major theme among creators is the dietary transition that occurs across various stages of post-surgical recovery. For many, this process begins with strict limitations in the immediate post-surgical period. These restrictions are understood by patients as necessary for healing and to prevent complications such as bowel obstructions.

*“As far as not being able to eat fruits and vegetables. So, this is not a long-term thing. This is for the month to 6 to 8 weeks”*—Video 19

*“Because your colon is removed and your small intestines are being, like, manipulated and moved around, they need to heal”*—Video 19

In the post-surgical period, patients often claimed to have followed “a low-residue, low-fibre” diet, which limits raw fruits and vegetables, seeds, nuts, and other “hard-to-digest” foods.

Three creators highlighted the association between dietary triggers and fear of small bowel obstruction in patients with an ileostomy, such as coconut and corn. These examples illustrate how certain fibrous foods are perceived to pose significant risk of small bowel obstruction in this patient group.

*“Last year I ate some coconut macaroons, which I will never be doing again, I’d spend a week in the hospital”*—Video 5

*“If you have an ileostomy, you need to stay away from certain kinds of foods like corn, because there’s the tendency for it to stay in the lumen of the intestine or the small bowel and cause an obstruction”*—Video 20

Some patients take a more nuanced approach to limiting fibrous foods, adjusting their intake based on how much time has passed since their surgery. These patients describe a crucial healing phase post-colectomy, where fibre and certain food groups are temporarily off-limits. They explain that a low-fibre, low-residue diet facilitates bowel healing and reduces inflammation. They emphasise how these dietary restrictions are often temporary rather than permanent. For example:

*“If you’re currently experiencing an IBD flare or you are post-surgery, do follow a low-residue diet, because obviously you’ve been through major surgery, and your bowel is experiencing major inflammation, so a low-residue diet is a lot more gentle on the digestive system…. I literally stuck solely for about 4 to 6 weeks to eating things like potato waffles, smiley faces, fish, chicken, eggs, really really well-cooked vegetables”*—Video 21

While there are significant dietary restrictions in the immediate post-surgical phase, some creators offer hope regarding longer-term dietary possibilities:

*“For the majority of those living with a stoma: you can eat whatever you want, and this could even be sort of way more than you had pre-surgery”*—Video 21

Here, the speaker contrasts the initial post-operative low-residue diet with the eventual reintroduction of a wider variety of foods, indicating a positive trajectory toward normalised eating.

### 3.2. Personalisation of Diet for Symptom and Hydration Management

A key message across patient videos is that there is no single “correct” post-surgical diet. Food tolerances vary significantly from person to person, and patients stress the importance of listening to one’s body rather than adhering to strict, uniform dietary rules. Patients promote self-experimentation and a personalised approach to food reintroduction. This involves closely monitoring symptoms, starting with familiar foods, and adjusting based on outcomes.

*“I slowly started to reintroduce new and different food types... do it one at a time”*—Video 21

*“Know the foods that make you feel good versus the ones that don’t”*—Video 38

Personalisation also extends to managing stoma output. Patients describe using food intentionally to alter stool consistency, such as choosing rice to thicken watery output or avoiding sugar to prevent high output.

*“I ended the night with just a plain bowl of rice to thicken up my output”*—Video 10

Meal-sharing content, such as “#whatIeatinaday” videos, appears to play a critical role for the community. These videos provide reassurance and practical insight for others navigating similar post-surgical challenges. This is particularly relevant considering the perceived limited availability of individualised guidance from healthcare providers. A variety of different meal plans and recipes were suggested in the videos, from ”stoma-friendly breakfasts,” of toast and peanut butter, high-protein matcha drinks, and outlines of meals that can be consumed across the whole day:

*“I started the day with an omelette with smoked salmon and pepper... and then for lunch time… red pesto salmon with steamed broccoli… Ahi tuna steak with salad. I hope these tips help you guys”*—Video 28

Hydration is a major concern post-surgery, both for those with an ileostomy and those with an ileo-anal pouch, as they explain that they are at higher risk of dehydration.

Many creators share personalised hydration routines, and recommend specific products based on experience and taste. Content creators also tended to prefer oral rehydration solution or electrolyte containing drinks as a superior choice to water in staying hydrated, and would explain the physiological basis for choosing these products.

*“I will always be on the brink of dehydration... I bring three drinks to work every day that make me feel a little less like I’m dying and a little more human”*—Video 30

Together, the above-mentioned strategies illustrate how patients actively manage their health post-surgery, adapting to a complex new way of living through meal planning, symptom monitoring and their hydration practices.

### 3.3. Emotional and Social Impact of Dietary Restrictions and Modifications

Dietary restrictions were described not only in the setting of symptom management, but as a negative impact on food-related quality of life. This reflects the symbolic importance of food in everyday life.

*“I’ve really been missing strawberries... but I can’t eat them right now because of the little seeds”*—Video 19

For some, food becomes a source of fear. The unpredictability of how the body might react to reintroduced foods creates hesitation and apprehension. The “trial and error” process of determining tolerances is daunting.

*“I don’t like the thought of the way to expand my diet might mean having to deal with extreme pain to figure out what works and doesn’t work. It is definitely super terrifying”*—Video 19

For one creator, fear of food translates into social withdrawal and a shrinking world. The narrowing of “safe foods” restricts not only diet but also social activities, such as eating out, or sharing meals with others. This consequently led to fears that these restrictions would be a permanent way of life. This highlights the psychological burden of dietary fear, contributing to isolation and reduced quality of life.

*“My life had gotten really small, and so had my list of safe foods. I was really fearful that my life was going to remain as small and as isolating as it was”*—Video 33

Despite these fears, not all creators view dietary expansion as threatening. Some promote a more confident, encouraging perspective, urging others not to let fear dictate their food choices.

*“All I can say is try the food. Don’t be afraid”*—Video 21

These counter-narratives offer hope and display resilience. They encourage a mindset of experimentation and learning, which can help mitigate anxiety and promote a sense of control.

## 4. Discussion

This thematic analysis offers valuable insights into the complex patient experience of managing diet following UC surgery. It identifies the specific domains where patients feel diet is most impactful. It also reveals the delicate balance between physical recovery and social wellbeing. These insights reinforce the necessity of patient-centred care that addresses the holistic burden of post-operative dietary modifications.

TikTok has become an increasingly popular and accessible platform for patients to connect with content seekers looking for information on IBD [17]. In our analysis, 75% of the short videos were made by patients with lived experience. This is similar to a previous study analysing TikTok content for ostomy management, where 100% of videos were created by individuals with IBD [18]. These findings highlight the clear desire in the IBD community to connect with other patients. They also suggest a potential gap of information provided by health professionals directed to patients with IBD. Given that patients with IBD spend 30–60 min each day on social media [19], increasing the availability of credible content from health professionals on platforms such as TikTok could be a useful method to reach the IBD audience.

Dietary restrictions followed by ”trial and error” reintroduction reflects the realities that UC patients face when managing their nutrition post-UC surgery. Therefore, turning to a global community for peer support and ideas might make sense for patients. In a recent survey of IBD patients, patients voiced reasons for seeking guidance online and on social media support groups due to a perceived lack of dietary information provided by healthcare professionals [20]. Interestingly, in our study, some of the most ”liked” videos were those explaining ”what I eat in a day,” and these engagement metrics reflect the content the community finds interesting. However, in a patient cohort that is already at a higher risk of disordered eating [21], there are very real risks of patients accessing algorithm-driven content that priorities popularity over accuracy. Medical misinformation is widespread on social media [22], and studies have shown that content with higher levels of misinformation often generates greater user engagement [23]. With TikTok’s increasing popularity, it may be prudent for clinicians, particularly those working with young people, to proactively discuss the types of nutrition information they receive online.

Restriction of fibre was a common strategy that patients used both during IBD flares and during the post-surgical recovery period. Reasons included allowing the bowel to “heal”, and preventing obstructions, which is largely based on habitual practice and experience-based, rather than evidence-based. In fact, the recent European Crohn’s and Colitis Organisation (ECCO) guidelines on dietary management of IBD [24] recommend diets low in bulking fibres only for those with structuring Crohn’s disease. Moreover, despite content creators using the terms ”low-residue” or ”low-fibre” there are no standardised definitions for these terms, raising concern that such messaging may inadvertently encourage unnecessary or prolonged restriction of fibre-containing foods among people with IBD.

The emotional aspect of dietary restrictions and social isolation that patients shared in their videos warrants greater clinical attention. Food-related quality of life, defined as the impact of food and eating on an individual’s physical, psychological, and social wellbeing [25], has been reported across multiple studies of IBD patients to be poor [26]. Our analysis illustrated the depth of this struggle. For one creator, fear of food translated into social withdrawal and a “shrinking world.” The narrowing of “safe foods” restricted not only their diet but also fundamental social activities, such as eating out or sharing meals with others. This led to profound anxieties that these restrictions would become a permanent way of life. Healthcare professionals should proactively acknowledge this and validate the psychological impact of dietary restrictions, incorporating psychosocial support or referrals to dietitians, psychologists, or support groups specialising in chronic gastrointestinal conditions [27]. These findings highlight the need for more holistic, multidisciplinary approaches that integrate psychological and dietary support.

Conversely, the presence of hopeful and empowering narratives among patients suggests opportunities for fostering resilience through peer support and positive reinforcement. Encouraging patients to adopt a gradual, fearless approach to reintroducing foods (backed by clinical safety netting) can mitigate anxiety and promote resilience [28].

Beyond its clinical implications, the digital behaviour observed in this study reflects a specialised dimension of consumer behaviour within the broader transition toward sustainable eating and health-oriented food systems. While sustainability is often viewed through an environmental lens, the long-term sustainability of dietary transitions for chronic disease populations depends on the accessibility of information and the psychological feasibility of dietary shifts. Our findings suggest that TikTok serves as a decentralised ”food policy” platform, where patients negotiate the conflict between restrictive medical diets and the desire for a sustainable, normalised social life. By crowdsourcing ”lived experience” data, these consumers are actively shaping a grassroots nutritional framework that prioritises the social and emotional sustainability of eating. These aspects are often overlooked in formal policy instruments. Recognising how digital health communication influences these specific consumer choices is essential for developing inclusive food policies that support both health outcomes and the long-term adherence to sustainable, health-conscious dietary patterns in vulnerable populations.

Our study has several limitations. First, our search strategy is subject to temporal bias. Because TikTok’s recommendation engine prioritises recency, the data extracted reflects discourse within the UC community at the specific time the search was conducted, and may disproportionately represent recently uploaded content rather than the historical breadth of videos on the platform. Second, screening only the “top 10” results for each search term introduces selection bias [29]. While this approach captured high-reach and highly engaged content, it likely excluded lower-performing videos that may contain unique or alternative perspectives which were less favoured by the platform’s engagement metrics.

Additionally, the results may be influenced by algorithmic and popularity bias. TikTok is not a neutral repository of information. Its recommendation system tends to amplify content that uses emotionally resonant framing or stereotypical narratives to drive engagement [30]. As a result, the themes identified in this study may reflect the types of content that perform well on the platform [31], rather than the full reality of the broader UC patient community.

Further limitations relate to the linguistic and cultural characteristics of the study sample. By including only English-language content, we excluded a significant segment of the UC community, most notably native Spanish speakers in the context of the United States. This represents a major cultural limitation, particularly given the high prevalence of Spanish-speaking TikTok users and the specific healthcare experiences of the Hispanic population [32,33]. This exclusion may also overlook critical cultural variations in alimentary habits and dietary management, which are central to the lived experience of UC patients. Furthermore, our sample may underrepresent the perspectives of older UC patients, whose dietary choices and lifestyle adjustments might differ from younger cohorts due to both cultural upbringing and disease longevity.

Participation bias is also a consideration. The data were derived from individuals who voluntarily share their experiences on social media, a group that may differ from the broader UC surgical population in terms of demographics, disease severity, or attitudes toward illness disclosure [34].

However, this digital medium may also address certain limitations inherent in traditional research methodologies. Recruitment for nutrition research often attracts more health-conscious participants, potentially limiting generalisability to the wider UC population [35]. Similarly, structured interviews or questionnaires conducted in clinical environments may constrain patient responses due to the formal setting [36]. In contrast, social media platforms such as TikTok provide an informal environment where patients can share experiences in real time as they unfold. This format allows for the expression of spontaneous, nuanced aspects of lived experience that may be difficult to capture through conventional research methods.

Consequently, although the sample represents a specific subset of the UC community, these data offer a unique and potentially more unfiltered perspective on post-surgical dietary experiences, insights that traditional clinical recruitment strategies may not readily capture.

Future studies should focus on longitudinal research that follows patients through the entire post-surgical dietary journey, to better understand temporal changes in diet tolerance, symptom management, and psychosocial impact.

Clinical trials investigating tailored dietary interventions and hydration protocols are also needed to establish evidence-based guidelines specific to the UC post-surgical population.

Furthermore, studies aimed at improving clinician communication strategies could help bridge the gap between clinical advice and patient experience.

## 5. Conclusions

In conclusion, our thematic analysis of TikTok content reveals that post-surgical UC patients utilise digital platforms to navigate complex dietary management while discussing three core themes: (1) a phased, adaptive dietary progression; (2) highly individualised approaches to symptom and hydration management; and (3) the negotiation of the profound emotional and social burdens of restrictive eating. These findings show that for many patients, the lived experience of dietary transition is as much about psychological resilience and social survival as it is about clinical nutrition. Healthcare providers should transition toward multidisciplinary models that validate these lived experiences and integrate formal guidance with the practical realities patients face.

Patient Perspective (Author O.K.): “Awakening after UC surgery will have been a traumatic experience for all patients. Patients not only have to cope with recovering from major surgery but also beginning to learn how to live with their altered anatomy! In both the pre- and post-operative periods, patients are given large amounts of information to understand about all the different aspects of their recovery after the surgery. Nutrition and food play a central role in patients’ quality of life, but the information can be overwhelming, particularly when patients are still physically and emotionally recovering! Even five years after my first surgery, I’m still learning how to best manage this aspect of my life. So, patients turn to TikTok and the online community to fill in those gaps on information they don’t fully receive from clinicians. Many of the videos were created by those with lived experiences, which on one hand is reassuring and incredibly empowering for patients, but they often lack clinical evidence, backed up with references on where that information has come from. This poses a huge risk for poor-quality information to be circulated amongst the online patient community and further reshared by other creators to be exposed to more patients.

In addition, several videos reflect the huge emotional and social weight that comes with diet, not just the practical advice about food choices. Fear of complications from certain foods, the anxiety associated with eating in social settings due to embarrassing symptoms like gas, odour, or unpredictable output, and the resulting sense of isolation patients experience, all have a profound impact on patients’ quality of life. I am fortunate to receive access to nutritional support from an IBD dietician but more is needed to address the psychological impact surrounding food and in providing support for all patients after UC surgery. If patients could access credible information online, education about the nutritional content available on platforms like TikTok, together with holistic support from clinicians on the wider issues surrounding food, they would not only feel safer in their dietary decisions but also be able to live a more fulfilling life.”

## Figures and Tables

**Table 1:** Video characteristics of videos included in thematic analysis.

Video Metrics	N (%) or Mean [Range]
**Total No. of videos**	**16 (100)**
**Video Duration (min:sec)**	**00:50 [00:15–04:35]**
**Creator/Account Type**	
- **Patient with lived experience**	**12 (75)**
- **Healthcare professional**	**2 (12)**
- **Patient & healthcare professional**	**1 (6)**
- **Stoma products**	**1 (6)**
**Country of Origin**	
- **USA**	**8 (50)**
- **UK**	**4 (25)**
- **Canada**	**1 (6)**
- **Morocco**	**1 (6)**
- **Unknown**	**2 (12)**
**Account engagement**	
- **Number of followers**	**19,960 [110–1.9M]**
- **Video likes**	**203 [16–1175]**

## Data Availability

The data underlying this study are available in the published article.

## References

[1] Quezada S.M., Cross R.K. (2012). Association of age at diagnosis and ulcerative colitis phenotype. Dig. Dis. Sci..

[2] Frolkis A.D., Dykeman J., Negrón M.E., Debruyn J., Jette N., Fiest K.M., Frolkis T., Barkema H.W., Rioux K.P., Panaccione R. (2013). Risk of surgery for inflammatory bowel diseases has decreased over time: A systematic review and meta-analysis of population-based studies. Gastroenterology.

[3] Gajendran M., Loganathan P., Jimenez G., Catinella A.P., Ng N., Umapathy C., Ziade N., Hashash J.G. (2019). A comprehensive review and update on ulcerative colitis. Dis. Mon..

[4] Christl S.U., Scheppach W. (1997). Metabolic consequences of total colectomy. Scand. J. Gastroenterol. Suppl..

[5] Spinelli A., Bonovas S., Burisch J., Kucharzik T., Adamina M., Annese V., Bachmann O., Bettenworth D., Chaparro M., Czuber-Dochan W. (2022). ECCO Guidelines on Therapeutics in Ulcerative Colitis: Surgical Treatment. J. Crohn’s Colitis.

[6] Peters V., Alizadeh B.Z., de Vries J.H., Dijkstra G., Campmans-Kuijpers M.J. (2019). Nutritional Assessment in Inflammatory Bowel Disease (IBD)-Development of the Groningen IBD Nutritional Questionnaires (GINQ). Nutrients.

[7] Czuber-Dochan W., Morgan M., Hughes L.D., Lomer M.C.E., Lindsay J.O., Whelan K. (2020). Perceptions and psychosocial impact of food, nutrition, eating and drinking in people with inflammatory bowel disease: A qualitative investigation of food-related quality of life. J. Hum. Nutr. Diet..

[8] Rhys-Jones D.R., Ghersin I., Argyriou O., Blackwell S., Lester J., Gibson P.R., Halmos E.P., Ardalan Z., Warusavitarne J., Sahnan K. (2025). A Quality Assessment and Evaluation of Credible Online Dietary Resources for Patients with an Ileoanal Pouch. J. Clin. Med..

[9] Lai Y., Liao F., He Z., Lai W., Zhu C., Du Y., Li Z. (2024). The status quo of short videos as a health information source of *Helicobacter pylori*: A cross-sectional study. Front. Public Health.

[10] Basch C.H., Hillyer G.C., Jaime C. (2020). COVID-19 on TikTok: Harnessing an emerging social media platform to convey important public health messages. Int. J. Adolesc. Med. Health.

[11] Denniss E., Lindberg R. (2025). Social media and the spread of misinformation: Infectious and a threat to public health. Health Promot. Int..

[12] The Lancet (2025). Health in the age of disinformation. Lancet.

[13] Blackburn M.R., Hogg R.C. (2024). #ForYou? the impact of pro-ana TikTok content on body image dissatisfaction and internalisation of societal beauty standards. PLoS ONE.

[14] Dondzilo L., Rodgers R.F., Dietel F.A. (2024). Association between engagement with appearance and eating related TikTok content and eating disorder symptoms via recommended content and appearance comparisons. Int. J. Eat. Disord..

[15] Munro E., Wells G., Paciente R., Wickens N., Ta D., Mandzufas J., Lombardi K., Woolard A. (2024). Diet culture on TikTok: A descriptive content analysis. Public Health Nutr..

[16] Braun V., Clarke V. (2006). Using thematic analysis in psychology. Qual. Res. Psychol..

[17] He Z., Wang Z., Song Y., Liu Y., Kang L., Fang X., Wang T., Fan X., Li Z., Wang S. (2023). The Reliability and Quality of Short Videos as a Source of Dietary Guidance for Inflammatory Bowel Disease: Cross-sectional Study. J. Med. Internet Res..

[18] Winders S., Yoo L., Conley S., Shapiro M., Pleasure Z.H., Kamp K. (2025). Inflammatory Bowel Disease on TikTok: Utilizing the Platform for Information on Ostomies, Advocacy, and Disease Management. Gastroenterol. Nurs..

[19] Reich J., Guo L., Groshek J., Weinberg J., Chen W., Martin C., Long M.D., Farraye F.A. (2019). Social Media Use and Preferences in Patients With Inflammatory Bowel Disease. Inflamm. Bowel Dis..

[20] Miglioretto C., Beck E., Lambert K. (2024). What do people with inflammatory bowel disease want to know about diet? The dietary information needs of people with inflammatory bowel disease and perceptions of healthcare providers. J. Hum. Nutr. Diet..

[21] Peters J.E., Basnayake C., Hebbard G.S., Salzberg M.R., Kamm M.A. (2022). Prevalence of disordered eating in adults with gastrointestinal disorders: A systematic review. Neurogastroenterol. Motil..

[22] Suarez-Lledo V., Alvarez-Galvez J. (2021). Prevalence of Health Misinformation on Social Media: Systematic Review. J. Med. Internet Res..

[23] Baghdadi J.D., Coffey K.C., Belcher R., Frisbie J., Hassan N., Sim D., Malik R.D. (2023). #Coronavirus on TikTok: User engagement with misinformation as a potential threat to public health behavior. JAMIA Open.

[24] Svolos V., Gordon H., Lomer M.C.E., Aloi M., Bancil A., Day A.S., Day A.S., Fitzpatrick J.A., Gerasimidis K., Gkikas K. (2025). European Crohn’s and Colitis Organisation consensus on dietary management of inflammatory bowel disease. J. Crohn’s Colitis.

[25] Hughes L.D., King L., Morgan M., Ayis S., Direkze N., Lomer M.C., Lindsay J.O., Whelan K. (2016). Food-related Quality of Life in Inflammatory Bowel Disease: Development and Validation of a Questionnaire. J. Crohn’s Colitis.

[26] Zhu W., Zhang Y., Wang L.D., Li J., Hou S. (2025). Factors influencing food-related quality of life in patients with inflammatory bowel disease: A systematic review. J. Eval. Clin. Pract..

[27] Mikocka-Walus A., Knowles S.R., Keefer L., Graff L. (2016). Controversies Revisited: A Systematic Review of the Comorbidity of Depression and Anxiety with Inflammatory Bowel Diseases. Inflamm. Bowel Dis..

[28] Xiong H., Zhang X., Zeng H., Xie S., Yi S. (2024). Experience of diet in patients with inflammatory bowel disease: A thematic synthesis of qualitative studies. J. Clin. Nurs..

[29] Rosen R., Vasiloudes V., Mhaskar R. (2024). The emergence of MedTok: A qualitative analysis of popular medical TikTok videos. Postgrad. Med. J..

[30] Zeng M., Grgurevic J., Diyab R., Roy R. (2025). #WhatIEatinaDay: The Quality, Accuracy, and Engagement of Nutrition Content on TikTok. Nutrients.

[31] Zenone M., Ow N., Barbic S. (2021). TikTok and public health: A proposed research agenda. BMJ Glob. Health.

[32] Velasco-Mondragon E., Jimenez A., Palladino-Davis A.G., Davis D., Escamilla-Cejudo J.A. (2016). Hispanic health in the USA: A scoping review of the literature. Public Health Rev..

[33] Pinkey A., Anzuman M., Desai M., Eiring A.M. (2025). Advances in the understanding of health disparities in the United States Hispanic population. J. Cancer Biol..

[34] Naslund J.A., Aschbrenner K.A., Marsch L.A., Bartels S.J. (2016). The future of mental health care: Peer-to-peer support and social media. Epidemiol. Psychiatr. Sci..

[35] Young L.M., Gauci S., Scholey A., White D.J., Pipingas A. (2020). Self-Selection Bias: An Essential Design Consideration for Nutrition Trials in Healthy Populations. Front. Nutr..

[36] Brédart A., Marrel A., Abetz-Webb L., Lasch K., Acquadro C. (2014). Interviewing to develop Patient-Reported Outcome (PRO) measures for clinical research: Eliciting patients’ experience. Health Qual. Life Outcomes.

